# A phase Ib study evaluating the recommended phase II dose, safety, tolerability, and efficacy of mivavotinib in combination with nivolumab in advanced solid tumors

**DOI:** 10.1002/cam4.6776

**Published:** 2024-03-19

**Authors:** Dejan Juric, Minal Barve, Ulka Vaishampayan, Desamparados Roda, Aitana Calvo, Noelia Martinez Jañez, Jose Trigo, Alastair Greystoke, R. Donald Harvey, Anthony J. Olszanski, Mateusz Opyrchal, Alexander Spira, Fiona Thistlethwaite, Begoña Jiménez, Jessica Huck Sappal, Karuppiah Kannan, Jason Riley, Cheryl Li, Cong Li, Richard C. Gregory, Harry Miao, Shining Wang

**Affiliations:** ^1^ Termeer Center for Targeted Therapies Massachusetts General Hospital Cancer Center Boston Massachusetts USA; ^2^ Medical Oncology Mary Crowley Cancer Research Dallas Texas USA; ^3^ Internal Medicine/Oncology, Karmanos Cancer Institute Wayne State University Detroit Michigan USA; ^4^ Department of Medical Oncology University Hospital Valencia Spain; ^5^ Medical Oncology Instituto de Investigación Sanitaria Gregorio Marañón Madrid Spain; ^6^ Department of Oncology Hospital Universitario Ramón y Cajal Madrid Spain; ^7^ Medical Oncology Hospital Universitario Virgen de la Victoria Málaga Spain; ^8^ Faculty of Medical Sciences Newcastle University Newcastle upon Tyne UK; ^9^ Hematology and Medical Oncology Winship Cancer Institute of Emory University Atlanta Georgia USA; ^10^ Department of Hematology/Oncology Fox Chase Cancer Center Philadelphia Pennsylvania USA; ^11^ Division of Oncology Washington University School of Medicine in St Louis St Louis Missouri USA; ^12^ Medical Oncology, Johns Hopkins School of Medicine Johns Hopkins University Baltimore Maryland USA; ^13^ Medical Oncology, Virginia Cancer Specialists US Oncology Research, NEXT Oncology Virginia Leesburg Virginia USA; ^14^ Medical Oncology The Christie NHS Foundation Trust and University of Manchester Manchester UK; ^15^ Precision and Translational Medicine Takeda Development Center Americas, Inc. (TDCA) Lexington Massachusetts USA; ^16^ Oncology Therapeutic Area Unit Takeda Development Center Americas, Inc. (TDCA) Lexington Massachusetts USA; ^17^ Gastroenterology Takeda Development Center Americas, Inc. (TDCA) Lexington Massachusetts USA; ^18^ Quantitative Clinical Pharmacology Takeda Development Center Americas, Inc. (TDCA) Lexington Massachusetts USA; ^19^ Statistical and Quantitative Sciences Takeda Development Center Americas, Inc. (TDCA) Lexington Massachusetts USA; ^20^ Clinical Development Takeda Development Center Americas, Inc. (TDCA) Lexington Massachusetts USA; ^21^ Takeda Oncology Clinical Science Takeda Development Center Americas, Inc. (TDCA) Lexington Massachusetts USA

**Keywords:** immunotherapy, mivavotinib, phase Ib, solid tumors, TNBC

## Abstract

Mivavotinib (TAK‐659/CB‐659), a dual SYK/FLT3 inhibitor, reduced immunosuppressive immune cell populations and suppressed tumor growth in combination with anti‐PD‐1 therapy in cancer models. This dose‐escalation/expansion study investigated the safety, pharmacokinetics, pharmacodynamics, and preliminary efficacy of mivavotinib plus nivolumab in patients with advanced solid tumors. Patients received oral mivavotinib 60–100 mg once‐daily plus intravenous nivolumab 3 mg/kg on days 1 and 15 in 28‐day cycles until disease progression or unacceptable toxicity. The dose‐escalation phase evaluated the recommended phase II dose (RP2D; primary endpoint). The expansion phase evaluated overall response rate (primary end point) at the RP2D in patients with triple‐negative breast cancer (TNBC). During dose‐escalation (*n* = 24), two dose‐limiting toxicities (grade 4 lipase increased and grade 3 pyrexia) occurred in patients who received mivavotinib 80 mg and 100 mg, respectively. The determined RP2D was once‐daily mivavotinib 80 mg plus nivolumab 3 mg/kg. The expansion phase was terminated at ~50% enrollment (*n* = 17) after failing to meet an ad hoc efficacy futility threshold. Among all 41 patients, common treatment‐emergent adverse events (TEAEs) included dyspnea (48.8%), aspartate aminotransferase increased, and pyrexia (46.3% each). Common grade ≥3 TEAEs were hypophosphatemia and anemia (26.8% each). Mivavotinib plasma exposure was generally dose‐proportional (60–100 mg). One patient had a partial response. Mivavotinib 80 mg plus nivolumab 3 mg/kg was well tolerated with no new safety signals beyond those of single‐agent mivavotinib or nivolumab. Low response rates highlight the challenges of treating unresponsive tumor types, such as TNBC, with this combination and immunotherapies in general.

**Trial registration ID:**

NCT02834247.

## 1 INTRODUCTION

Targeting the tumor immune microenvironment (TIME) is an increasingly important area of research for cancer therapies. Cross talk between cancer cells and immune cells in the TIME establishes a pro‐tumoral environment, leading to impaired immune surveillance and tumor immune escape.^1^ Many tumors suppress the activity of immune cells via amplification of direct signaling pathways such as the programmed death‐1 receptor (PD‐1)/programmed death ligand‐1 (PD‐L1) pathway, which attenuates T‐cell activation in the TIME^2^ and promotes proliferation of regulatory T (Treg) cells.^3^ Additionally, tumors promote immune suppression indirectly via transformation of hematopoietic progenitor cells into immune‐suppressing myeloid‐derived suppressor cells (MDSCs),^1^ which infiltrate tumors and inhibit a range of proinflammatory cells and/or promote anti‐inflammatory cells.^1^

Antibody blockade of immune checkpoint molecules such as PD‐1 and cytotoxic T lymphocyte‐associated protein 4 (CTLA‐4) has produced substantial clinical effects across various tumor types.^4^ However, these checkpoint inhibitors (CPIs) are only curative in a small number of cases that are limited to specific cancer types.^4^ The presence of MDSCs in the TIME has been identified as largely responsible for the limited clinical outcomes of immunotherapy^1^, ^5^, ^6^; however, there is evidence that reprogramming or eliminating MDSCs can improve response to anti‐PD‐1 treatment in solid tumors.^5^, ^7^ Consequently, investigations into new treatment strategies are increasingly combining immunotherapies, such as CPIs, with therapies that deplete the population or activity of MDSCs.^6^, ^8^

Nivolumab is a PD‐1 inhibitor^9^ that restores endogenous anticancer responses by abrogating PD‐1 pathway‐mediated T‐cell inhibition.^10^ Nivolumab is approved as a single‐agent or in combination with ipilimumab and other agents to treat a variety of malignancies; these approvals span the United States and Europe.^11^, ^12^ Research is underway into combination therapies that may enhance the antitumor activity of nivolumab with several classes of investigational agents.^13^, ^14^, ^15^, ^16^

Mivavotinib (TAK‐659/CB‐659) is an investigational, oral, selective, and potent dual inhibitor of spleen tyrosine kinase (SYK) and FMS‐like tyrosine kinase 3 (FLT3).^17^, ^18^ FLT3 and SYK are key molecules in multiple signaling pathways in MDSCs and Treg cells.^8^, ^19^, ^20^, ^21^ Furthermore, SYK signaling appears to be essential for the polarization of macrophages to the pro‐tumorigenic tumor‐associated macrophage (TAM) phenotype.^8^ Preclinical studies in multiple syngeneic or xenograft models of solid tumors, including triple‐negative breast cancer (TNBC), showed that mivavotinib reduces population numbers of immunosuppressive cells, including MDSCs and Treg cells.^18^, ^22^, ^23^ The presence of MDSCs in the tumor microenvironment is involved in tumor growth and metastasis.^1^, ^4^ In a syngeneic colon cancer model, mivavotinib in combination with anti‐PD‐1 therapy resulted in a loss of MDSCs, complete and durable tumor growth suppression, and prolonged tumor‐free survival.^18^ Mivavotinib in combination with nivolumab may therefore have therapeutic potential for solid tumors associated with MDSC‐mediated tumor immunosuppression,^22^, ^23^, ^24^ such as TNBC,^25^ some types of non‐small cell lung cancer (NSCLC),^26^ and head and neck squamous cell carcinoma (HNSCC).^27^

This phase Ib study was initiated to evaluate the safety, tolerability, and preliminary efficacy of mivavotinib in combination with nivolumab for the treatment of patients with advanced solid tumors.

## 2 PATIENTS AND METHODS

### 2.1 Preclinical studies

Female BALB/c mice (JAX) were inoculated with 0.2 × 10^6^ CT26 tumor cells in the left hind flank 7 days prior to randomization, allowing tumors to grow to 75–100 mm^3^ by the time of treatment initiation. Mice were treated with either vehicle, 60 mg/kg mivavotinib orally once daily (QD) for 14 days (a higher dose than used in the clinical study), anti‐PD‐1 10 mg/kg twice‐weekly intraperitoneally for five doses (0, 2, 6, 8, and 11), or mivavotinib in combination with anti‐PD‐1. Mice surviving combination therapy (15/15 [100%]) were followed for tumor progression. Tumor‐free mice were rechallenged with CT26 tumor cells in the right flank, except for a naïve cohort as a control. To assess M1 and M2 macrophages, tumors were harvested, digested enzymatically, and assayed via flow cytometry.

### 2.2 Phase Ib study design

This phase Ib, open‐label, multicenter, dose‐escalation, and expansion study (NCT02834247) recruited patients with advanced solid tumors at 13 sites in the United States, United Kingdom, and Spain. The primary end points were maximum tolerated dose (MTD) or recommended phase II dose (RP2D) of mivavotinib combined with nivolumab (dose‐escalation phase) and overall response rate (ORR) in specific tumor types (expansion phase). Secondary end points included: mivavotinib pharmacokinetics (PKs); proportion of patients with treatment‐emergent adverse events (TEAEs); discontinuations due to TEAEs; disease control rate (proportion of patients with a response or stable disease [SD]); duration of response (DOR); progression‐free survival (PFS); and overall survival (OS). Changes in biomarkers in tumors and tumor microenvironments, such as tumor‐infiltrating lymphocytes, MDSCs, and cytokine/chemokine receptors, identified by analysis of paired tumor biopsies was an exploratory end point.

Patients received oral mivavotinib QD in combination with nivolumab 3 mg/kg administered intravenously (IV) as a 60‐min infusion once every 2 weeks (Q2W; days 1 and 15) in 28‐day cycles (patients who received 2 weeks of mivavotinib monotherapy before starting combination treatment, received their first nivolumab infusion on cycle 1 day 15). Mivavotinib dose‐escalation followed a standard 3 + 3 design with a starting dose of 60 mg QD and maximally administered dose of 100 mg QD, based on the starting dose and MTD of single‐agent mivavotinib in a first‐in‐human phase I study of patients with solid tumors and lymphoma.^28^ Evaluation of intermediate mivavotinib dose levels between 60 and 100 mg or below the starting dose of 60 mg was permissible based on available safety, tolerability, and preliminary PK and efficacy data.^28^ The MTD of mivavotinib in combination with nivolumab was determined based on dose‐limiting toxicities (DLTs; defined in the Data S1) in cycle 1, and the RP2D was determined based on available safety, tolerability, and preliminary PK and efficacy data.

Following identification of the mivavotinib RP2D in combination with nivolumab, patients with TNBC were enrolled to the expansion phase. The expansion phase was planned to enroll three cohorts of patients with TNBC, NSCLC, and HNSCC; however, due to the changing treatment landscape and resource prioritization, the sponsor in collaboration with the study investigators decided to limit enrollment during expansion to patients with metastatic TNBC only. Patients were planned to receive mivavotinib QD (at the MTD/RP2D) in 28‐day cycles plus nivolumab 3 mg/kg Q2W starting either on day 1 or on day 15 (2:1 ratio) of cycle 1. The latter group received single‐agent mivavotinib for 2 weeks to enable assessment of the pharmacodynamic effects of mivavotinib alone.

### 2.3 Patients

For dose‐escalation, patients had to be ≥18 years old with previously treated (≥1 prior therapy), histologically confirmed, advanced solid tumors (and radiographically or clinically evaluable disease), and an Eastern Cooperative Oncology Group performance status (ECOG PS) of 0 or 1, and for whom no effective therapeutic options were available based on investigator assessment. In expansion, patients had to be ≥18 years old with histologically confirmed, metastatic TNBC, measurable disease per Response Evaluation Criteria in Solid Tumors (RECIST) version 1.1, an ECOG PS of 0 or 1, and have received 1–3 prior lines of chemotherapy for metastatic disease, with disease progression on their last regimen (neoadjuvant/adjuvant treatment did not count as a prior line; prior treatment must have included an anthracycline and/or taxane in the neoadjuvant, adjuvant, or metastatic setting, unless contraindicated). One third of patients in each expansion cohort were required to have tumors accessible for core or excisional biopsy. Patients were excluded if they had received prior therapy with any T‐cell co‐stimulation agents or inhibitors of checkpoint pathways (patients in the dose‐escalation phase were allowed prior treatment with marketed CPIs and six response‐evaluable patients planned in each of the expansion cohorts were allowed prior treatment with marketed/investigational CPIs). Full eligibility criteria are in the Data S1.

### 2.4 Assessments

Toxicity was evaluated using the Medical Dictionary for Regulatory Activities (version 22.0) and graded according to the National Cancer Institute Common Terminology Criteria for Adverse Events version 4.03. Adverse events (AEs) were monitored from provision of informed consent through 28 days after the last dose of study treatment or start of subsequent anticancer therapy, whichever occurred first.

Disease assessment was by computed tomography scan with contrast (or magnetic resonance imaging if clinically indicated), relevant tumor markers (e.g., CA125, prostate‐specific antigen, CA19.9, carcinoembryonic antigen), and physical assessment at screening, between day 22 and 29 of cycles 2, 4, and 6, every third cycle thereafter, and during follow‐up. Response was assessed by investigators according to RECIST version 1.1.^29^ A confirmatory scan was conducted approximately 4 weeks from the previous scan for all patients with an objective response. Patients were followed up every 2 months from the last dose of study drug until progressive disease (PD) for a maximum of 6 months for PFS and 12 months for OS.

During dose‐escalation, blood samples for the assessment of mivavotinib PK were collected pre‐dose (within 1 h) and 0.5, 1, 2, 4, 8, and 24 h post‐dose on days 1 and 15 of cycle 1. In the expansion phase, blood samples were collected on days 1, 8, and 15 of cycle 1 and on day 1 of cycles 2–4 for population PK analyses. Mivavotinib plasma concentrations were determined by liquid chromatography/tandem mass spectrometry assay methods validated over the concentration range 1–1000 ng/mL. PK parameters for mivavotinib were estimated using noncompartmental methods.

For analysis of the pharmacodynamic effects of mivavotinib on the TIME, paired tumor biopsies were obtained from patients with TNBC at screening and on day 15 of cycle 1 (prior to initial nivolumab) from patients in the expansion phase who did not receive the day 1 dose of nivolumab. MultiOmyx™ technology was utilized to evaluate the expression of a panel of 14 markers: CD3, CD4, CD8, CD45RO, FOXP3, CD56, CD68, CD20, Granzyme B, CTLA‐4, PD‐1, PD‐L1, Ki67, and tumor segmentation marker PanCK. Details of staining can be found in the Data S1. Individual cell classification results were combined to generate co‐expression summaries and to compute spatial distribution statistics for phenotypes of interest.

Peripheral blood from patients was collected by standard venipuncture techniques at screening and prior to dosing on cycle 1 day 15, cycle 3 day 1, and at the end of treatment. Samples were shipped overnight to Primity Bio (Fremont, CA) at ambient temperature for flow cytometry analysis performed on an LSR II (Becton Dickson) to assess change in T cells, B cells, natural killer (NK) cells, dendritic cells, MDSCs, and monocytes, and analyzed with proprietary software (Primity Bio). See the Data S1 for a description of markers.

### 2.5 Statistical analysis

Estimated planned enrollment in the dose‐escalation phase was 12–18 DLT‐evaluable patients. Study populations are described in the Data S1. Estimated planned enrollment in the expansion phase was ~36 patients with a target of 30 response‐evaluable patients. Time‐to‐event variables (DOR, PFS, and OS) were estimated using Kaplan–Meier methodology.

The reported findings represent the final analysis of this study. An ad hoc futility analysis was conducted in the TNBC expansion cohort following enrollment of ~50% of the estimated sample size. The null hypothesis had a response rate of ≤20% and the alternative hypothesis was a rate of ≥40% for patients naïve to anti‐PD/PD‐L1 and any other immune‐directed antitumor therapies. The futility analysis revealed insufficient antitumor activity to warrant continued recruitment, and the study was terminated early by the sponsor.

## 3 RESULTS

### 3.1 Preclinical studies

**FIGURE 1:**
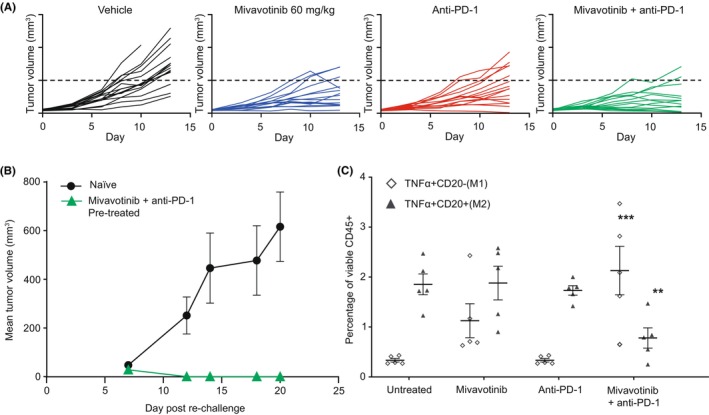
(A) Tumor growth inhibition curves for single and combination therapy of mivavotinib and anti‐PD‐1 in the CT26 syngeneic mouse tumor model. (B) Tumor rechallenge. (C) Pharmacodynamic assessment. M1/M2, M1/M2 macrophages; PD‐1, programmed death‐1 receptor; TNFa, tumor necrosis factor alfa. *p ≤ 0.05, **p ≤ 0.01, and ***p ≤ 0.001. One‐Way ANOVA with Dunnett's multiple comparisons test, with a single pooled variance.

Treatment with mivavotinib combined with anti‐PD‐1 in a CT26 syngeneic tumor model in female BALB/c mice resulted in complete tumor regression in 11/15 mice (73.3%) (Figure 1A); mice were tumor free for ≥100 days posttreatment. When these mice were rechallenged with CT26 tumor cells, no tumors were formed (Figure 1B) suggesting a memory T‐cell effect was established from the initial treatment period. Treatment with mivavotinib in combination with anti‐PD‐1 also resulted in a significant increase in the M1 macrophage population and a decrease in the M2 macrophage population (Figure 1C).

### 3.2 Phase Ib clinical study

Overall, 41 patients were enrolled, 24 with advanced solid tumors in the dose‐escalation phase, and 17 with TNBC in the expansion phase. In dose‐escalation, patients received mivavotinib QD at doses of 60 mg (*n* = 8), 80 mg (*n* = 11), and 100 mg (*n* = 5); all patients also received nivolumab 3 mg/kg IV Q2W. In the expansion phase in TNBC patients, 17 patients received mivavotinib 80 mg QD in combination with nivolumab 3 mg/kg, including five patients who received mivavotinib alone for the first 2 weeks. Patient demographics and baseline characteristics are shown in Table 1. Thirty‐two (78.0%) patients were female and 32 (78.0%) were white. Median age was 53.0 years (range: 23–76). The most common cancer types were breast (*n* = 22; 53.7%), ovarian (*n* = 3; 7.3%), lung (*n* = 2; 4.9%), and pancreatic (*n* = 2; 4.9%). Most patients (*n* = 36; 87.8%) had stage IV disease at study entry, and most (*n* = 36; 87.9%) had received ≥2 prior lines of therapy. Among the 17 patients with TNBC enrolled to receive mivavotinib 80 mg QD in combination with nivolumab 3 mg/kg in the expansion phase, 16 were naïve to anti‐PD‐1/PD‐L1 treatment.

**TABLE 1:** Patient demographics and baseline characteristics, overall and by study phase and dose level.

	Dose‐escalation				Expansion	
	Mivavotinib	Mivavotinib	Mivavotinib	Total	Mivavotinib	Total
	60 mg QD	80 mg QD	100 mg QD		80 mg QD	
	(*n* = 8)	(*n* = 11)	(*n* = 5)	(*N* = 24)	(*n* = 17)	(*N* = 41)
Median age, years (range)	56.0 (48–76)	59.0 (23–69)	50.0 (39–75)	56.0 (23–76)	50.0 (33–63)	53.0 (23–76)
Age category, *n* (%)						
18–64 years	7 (87.5)	9 (81.8)	3 (60.0)	19 (79.2)	17 (100)	36 (87.8)
≥65 years	1 (12.5)	2 (18.2)	2 (40.0)	5 (20.8)	0	5 (12.2)
Gender, *n* (%)						
Female	5 (62.5)	8 (72.7)	2 (40.0)	15 (62.5)	17 (100)	32 (78.0)
Male	3 (37.5)	3(27.3)	3 (60.0)	9 (37.5)	0	9 (22.0)
Race, *n* (%)						
White	7 (87.5)	9 (81.8)	4 (80.0)	20 (83.3)	12 (70.6)	32 (78.0)
Black/African American	1 (12.5)	0	1 (20.0)	2 (8.3)	2 (11.8)	4 (9.8)
Asian	0	2 (18.2)	0	2 (8.3)	1 (5.9)	3 (7.3)
Missing	0	0	0	0	2 (11.8)	2 (4.9)
ECOG PS, *n* (%)						
0	1 (12.5)	2 (18.2)	1 (20.0)	4 (16.7)	8 (47.1)	12 (29.3)
1	7 (87.5)	9 (81.8)	4 (80.0)	20 (83.3)	9 (52.9)	29 (70.7)
Cancer types, *n* (%)						
Breast	1 (12.5)	3 (27.3)	1 (20.0)	5 (20.8)	17 (100)	22 (53.7)
Ovarian	1 (12.5)	1 (9.1)	1 (20.0)	3 (12.5)	0	3 (7.3)
Colon	0	1 (9.1)	1 (20.0)	2 (8.3)	0	2 (4.9)
Lung	1 (12.5)	1 (9.1)	0	2 (8.3)	0	2 (4.9)
Pancreatic ductal	0	2 (18.2)	0	2 (8.3)	0	2 (4.9)
Bile duct	0	1 (9.1)	0	1 (4.2)	0	1 (2.4)
Cervical	0	1 (9.1)	0	1 (4.2)	0	1 (2.4)
Colorectal	0	1 (9.1)	0	1 (4.2)	0	1 (2.4)
Head and neck squamous cell	0	0	1 (20.0)	1 (4.2)	0	1 (2.4)
Liver and intrahepatic bile duct	1 (12.5)	0	0	1 (4.2)	0	1 (2.4)
Prostatic	1 (12.5)	0	0	1 (4.2)	0	1 (2.4)
Sarcoma	1 (12.5)	0	0	1 (4.2)	0	1 (2.4)
Signet cell ring	1 (12.5)	0	0	1 (4.2)	0	1 (2.4)
Squamoid eccrine ductal	1 (12.5)	0	0	1 (4.2)	0	1 (2.4)
Unknown	0	0	1 (20.0)	1 (4.2)	0	1 (2.4)
Disease stage at study entry, *n* %						
I	0	0	0	0	1 (5.9)	1 (2.4)
II	1 (12.5)	0	0	1 (4.2)	1 (5.9)	2 (4.9)
III	0	0	0	0	2 (11.8)	2 (4.9)
IV	7 (87.5)	11 (100)	5 (100)	23 (95.8)	13 (76.5)	36 (87.8)
Number of prior lines of therapy, *n* (%)						
1	1 (12.5)	0	1 (20.0)	2 (8.3)	3 (17.6)	5 (12.2)
2	1 (12.5)	2 (18.2)	0	3 (12.5)	6 (35.3)	9 (22.0)
≥3	6 (75.0)	9 (81.8)	4 (80.0)	19 (79.2)	8 (47.1)	27 (65.9)

Abbreviations: ECOG PS, Eastern Cooperative Oncology Group performance status; QD, once daily.

All patients had discontinued treatment at the data cutoff, primarily due to disease progression in 25 patients (61.0%), and TEAEs in 10 patients (24.4%) (Figure S1).

### 3.3 Dose‐limiting toxicities and MTD/RP2D determination

Of the 24 patients in the dose‐escalation phase, 19 were DLT‐evaluable. No DLTs were recorded for six patients dosed at 60 mg, one of nine patients dosed at 80 mg had a DLT of grade 4 lipase increased, which was asymptomatic, and one of four patients dosed at 100 mg had a DLT of grade 3 pyrexia during cycle 1. Based on the DLT and long‐term tolerability concerns at 100 mg reported in a separate single‐agent study, the decision was made to stop expanding at 100 mg per the 3 + 3 design and instead enroll additional patients at 80 mg. The RP2D for mivavotinib plus nivolumab was determined as 80 mg QD based on evaluation of long‐term safety, tolerability, and preliminary response data, and the MTD was not defined.

### 3.4 Safety

All 41 patients received ≥1 dose of either study drug and were included in the safety population. Patients received a median of 2.0 cycles (range: 1.0–12.0) of mivavotinib in the dose‐escalation phase and 2.0 cycles (range: 1.0–3.0) in the expansion phase. For nivolumab, patients received a median of 2.0 cycles in both dose‐escalation (range, 1.0–12.0) and expansion (range: 0.0–3.0). All patients experienced ≥1 TEAE (Table S1). The most common TEAEs overall were dyspnea (48.8%), aspartate aminotransferase (AST) increased, pyrexia (46.3% each), fatigue, and diarrhea (43.9% each) (Table 2). Mivavotinib‐related TEAEs were reported in 27 (65.9%) patients, nivolumab‐related TEAEs were reported in 16 (39.0%) patients, and TEAEs considered related to both agents in 21 (51.2%) patients (Table S1). Most frequent (occurring in >10% of patients) TEAEs related to mivavotinib were pyrexia, amylase increased, and AST increased (*n* = 8, 19.5% each), followed by lipase increased (*n* = 6, 14.6%), diarrhea, and rash (*n* = 5, 12.2% each). The most common (occurring in >5% of patients) TEAEs related to nivolumab were rash (*n* = 5, 9.8%), alanine aminotransferase increased, and AST increased (*n* = 3, 7.3% each). The most common TEAEs related to both mivavotinib and nivolumab were diarrhea (*n* = 6; 14.6%), nausea, AST increased, and fatigue (*n* = 5, 12.2% each). Increases in alanine aminotransferase, AST, amylase, and lipase were asymptomatic, reversible, and clinically insignificant.

**TABLE 2:** Most common treatment‐emergent adverse events (≥20% of patients overall).

	Dose‐escalation				Expansion	
	Mivavotinib	Mivavotinib	Mivavotinib		Mivavotinib	
	60 mg QD	80 mg QD	100 mg QD	Total	80 mg QD	Total
	(*n* = 8)	(*n* = 11)	(*n* = 5)	(*N* = 24)	(*n* = 17)	(*N* = 41)
	*n* (%)	*n* (%)	*n* (%)	*n* (%)	*n* (%)	*n* (%)
Dyspnea	3 (37.5)	6 (54.5)	1 (20.0)	10 (41.7)	10 (58.8)	20 (48.8)
AST increased	2 (25.0)	6 (54.5)	4 (80.0)	12 (50)	7 (41.2)	19 (46.3)
Pyrexia	3 (37.5)	4 (36.4)	3 (60.0)	10 (41.7)	9 (52.9)	19 (46.3)
Diarrhea	4 (50.0)	6 (54.5)	2 (40.0)	12 (50.0)	6 (35.3)	18 (43.9)
Fatigue	5 (62.5)	5 (45.5)	3 (60.0)	13 (54.2)	5 (29.4)	18 (43.9)
ALT increased	2 (25.0)	6 (54.5)	3 (60.0)	11 (45.8)	5 (29.4)	16 (39.0)
Anemia	3 (37.5)	6 (54.5)	2 (40.0)	11 (45.8)	4 (23.5)	15 (36.6)
Hypophosphatemia	2 (25.0)	7 (63.6)	1 (20.0)	10 (41.7)	4 (23.5)	14 (34.1)
Amylase increased	0.0	6 (54.5)	3 (60.0)	9 (37.5)	4 (23.5)	13 (31.7)
Cough	3 (37.5)	6 (54.5)	1 (20.0)	10 (41.7)	3 (17.6)	13 (31.7)
Hypokalemia	2 (25.0)	8 (72.7)	0	10 (41.7)	3 (17.6)	13 (31.7)
Nausea	5 (62.5)	2 (18.2)	0.0	7 (29.2)	4 (23.5)	11 (26.8)
Constipation	2 (25.0)	4 (36.4)	0.0	6 (25.0)	4 (23.5)	10 (24.4)
Rash	2 (25.0)	2 (18.2)	2 (40.0)	6 (25.0)	4 (23.5)	10 (24.4)
Edema peripheral	3 (37.5)	2 (18.2)	0.0	5 (20.8)	5 (29.4)	10 (24.4)
Vomiting	4 (50.0)	4 (36.4)	1 (20.0)	9 (37.5)	0	9 (22.0)

*Note*: MedDRA version 22.0 was used for coding TEAEs. TEAEs were defined as any AEs that occurred after the administration of the first dose of study drug and through 28 days after the last dose of study drug or until the start of subsequent anticancer therapy. In the expansion group, 12 patients received mivavotinib and nivolumab from day 1, and five patients received only mivavotinib on days 1–14, plus nivolumab from day 15.

Abbreviations: AE, adverse event; ALT, alanine aminotransferase; AST, aspartate aminotransferase; MedDRA, Medical Dictionary for Regulatory Activities; QD, once daily; TEAE, treatment‐emergent adverse event.

Grade ≥3 TEAEs occurred in 34 (82.9%) patients (Table S1). The grade ≥3 TEAEs reported in >10% of patients overall were hypophosphatemia (*n* = 11, 26.8%), anemia (*n* = 11, 26.8%), lipase increased (*n* = 6, 14.6%), fatigue (*n* = 6, 14.6%), AST increased, hypokalemia, and pneumonitis (*n* = 5, 12.2% each). Grade ≥3 TEAEs reported in four or more patients that were mivavotinib‐related were lipase increased and hypophosphatemia (*n* = 4, 9.8% each). The only nivolumab‐related grade ≥3 TEAE reported in more than one patient was pneumonitis. Grade ≥3 TEAEs related to both mivavotinib and nivolumab were, most commonly, pneumonitis (*n* = 3, 7.3%), rash, and fatigue (*n* = 2, 4.9% each). Serious TEAEs were reported in 29 (70.7%) patients (Table S1); the most common were pyrexia (*n* = 7, 17.1%), pneumonitis (*n* = 4, 9.8%), dyspnea, and sepsis (*n* = 3, 7.3% each). Ten (24.4%) patients discontinued both mivavotinib and nivolumab due to TEAEs, and a further one (2.4%) and three (7.3%) patients discontinued mivavotinib and nivolumab alone, respectively, due to TEAEs (Table S1). The only TEAE leading to study drug discontinuation in more than one patient overall was pneumonitis (*n* = 4, all in the expansion phase), which was considered related to both drugs. Other TEAEs that led to patients discontinuing both mivavotinib and nivolumab were ataxia, amylase increased, lipase increased, troponin increased, flushing, dry mouth, myocarditis, and dyspnea (*n* = 1 each). Twenty‐four patients died during the study or during follow‐up, 16 patients in the dose‐escalation phase and eight in the expansion phase. Twenty patients died from disease progression or causes related to the disease under study. Three patients died due to AEs of cardiac arrest, sepsis, and respiratory failure, respectively, considered not related to either mivavotinib or nivolumab; cause of death was unknown in one patient. Seven patients died within 28 days of the last dose of mivavotinib, including four who died from disease progression or causes related to the disease under study and the three who died due to AEs.

### 3.5 Pharmacodynamic markers

**FIGURE 2:**
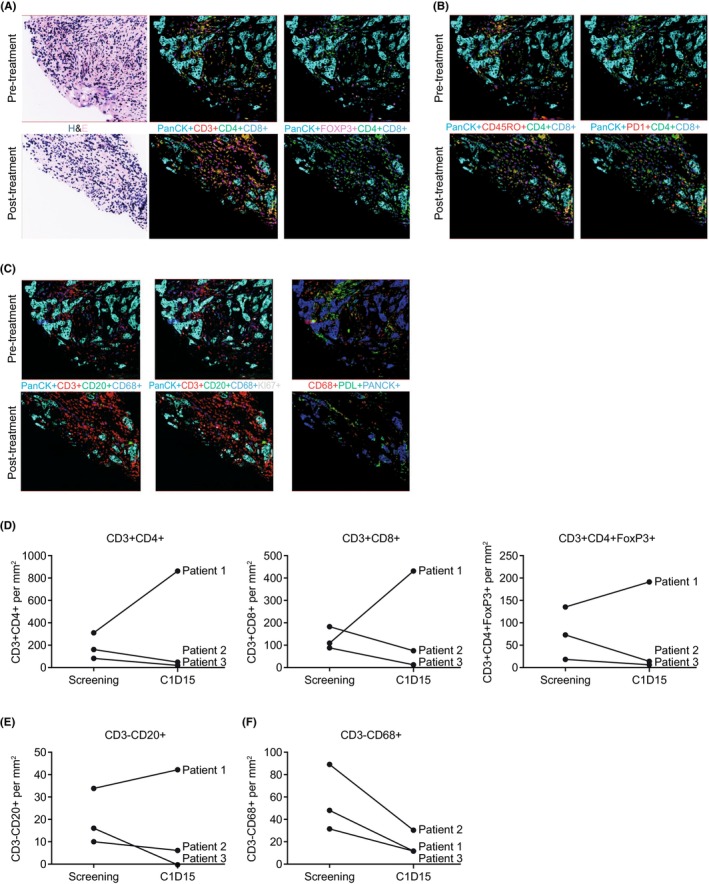
Change from screening to day 15 in (A) CD4, CD8 and Treg cells expression, (B) memory T cells and PD‐L1 positivity, (C) monocytes, B cells and PD‐L1 expression, assessed by multiplex fluorescence immunohistochemistry of paired tumor biopsies from one patient with TNBC treated with single‐agent mivavotinib. Change from screening to day 15 in (D) tumoral T cells, (E) monocytes, NK cells, and B cells, and (F) CD68+ monocytes/macrophages, assessed by multiplex fluorescence immunohistochemistry of paired tumor biopsies from three TNBC patients in the expansion phase. C, cycle; D, day; H&E, hematoxylin and eosin staining; NK, natural killer; PanCK, pan‐cytokeratin; PD‐L1, programmed death‐ligand 1; TNBC, triple‐negative breast cancer; Treg, regulatory T cells.

Paired tumor biopsies were obtained from three TNBC patients at screening and on day 15 of cycle 1, prior to the start of nivolumab dosing, to evaluate the pharmacodynamic effects of mivavotinib in terms of immune cell population levels. Figure 2 shows changes in immunohistochemical staining for CD4, CD8, and Treg cells expression (Figure 2A), memory T cells and PD‐L1 positivity (Figure 2B), and monocytes, B cells and PD‐L1 expression (Figure 2C) in paired biopsies from a single patient with TNBC treated with single‐agent mivavotinib. Two of the three biopsied patients received mivavotinib alone on days 1–14 and in combination with nivolumab from day 15 onward; the third patient paused mivavotinib treatment on day 12 and resumed mivavotinib monotherapy on day 23 but did not receive nivolumab. Notably, expression of CD3 + CD4+ T‐helper cells, CD3 + CD4 + FoxP3+ Treg cells, CD3 + CD8+ cytotoxic T cells (Figure 2D), and CD20+ B cells (Figure 2E) decreased from screening to day 15 of cycle 1 in two patients but increased in the patient who paused treatment. A decrease in CD68+ macrophages between screening and cycle 1 day 15 was observed in tumor tissue from all three patients (Figure 2F). All the three patients had PD by or at the start of cycle 2.

**FIGURE 3:**
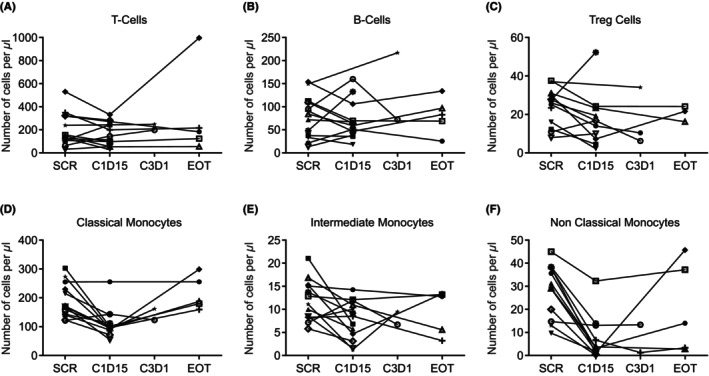
Changes in peripheral T cells, B cells, and monocytes in patients treated with mivavotinib and nivolumab assessed by flow cytometry. C, cycle; D, day; EOT, end of treatment; SCR, screening; Treg, regulatory T cells.

Changes in immune cell population in response to mivavotinib plus nivolumab were assessed in peripheral blood samples from 13 patients. While the numbers of CD3+ T cells (Figure 3A) and CD20+ B cells (Figure 3B) showed modest changes between screening and day 15 of cycle 1, CD4 + CD25 + CD127(low) Treg cells showed a downward trend (Figure 3C). The levels of circulating classical (CD14 + CD16‐; Figure 3D), intermediate (CD14 + CD16+; Figure 3E), and nonclassical (CD14[low]CD16+; Figure 3F) monocytes were reduced in most patients, suggesting a peripheral pharmacodynamic impact of mivavotinib upon the monocyte lineage. These results are consistent with the paired biopsy results in which macrophages were decreased in response to mivavotinib treatment. It is unclear whether mivavotinib treatment results in death of the monocyte lineage or blocks the development of new monocytes.

### 3.6 Pharmacokinetics

**FIGURE 4:**
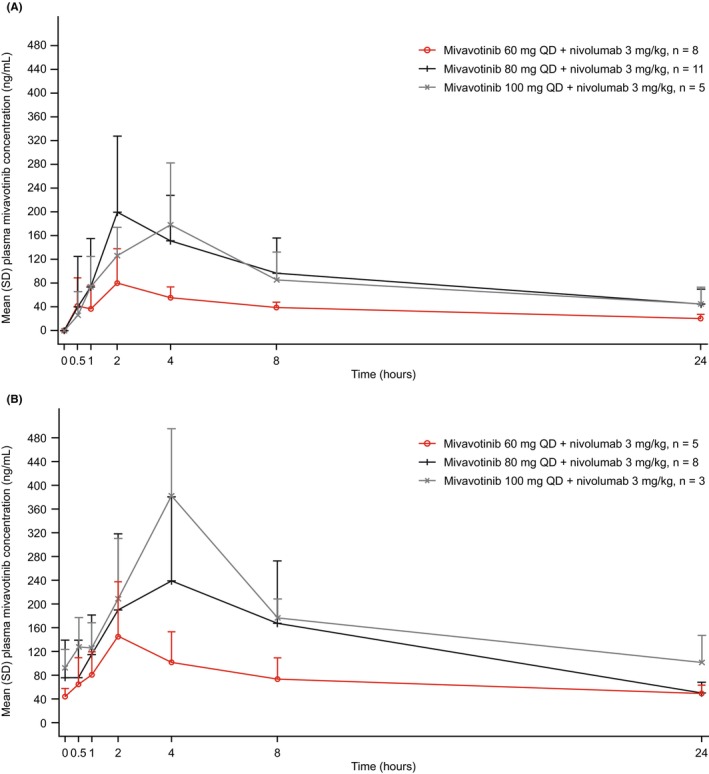
Mean (SD) plasma mivavotinib concentration–time profiles after a single‐dose of mivavotinib on (A) day 1 of cycle 1 and after multiple‐dose administration on (B) day 15 of cycle 1 in patients receiving mivavotinib at 60 mg, 80 mg, or 100 mg QD in combination with nivolumab 3 mg/kg. QD, once daily; SD, standard deviation. Plasma concentrations below the lower limit of quantification of the assay (<0.5 ng/mL) were recorded as zero.

All 24 patients in the dose‐escalation phase were PK‐evaluable. The mean (±standard deviation) plasma concentration–time profiles of mivavotinib on days 1 and 15 of cycle 1 are shown in Figure 4. Following single‐dose administration (day 1, *n* = 24) and multiple‐dose (day 15, *n* = 16) administration, mivavotinib demonstrated rapid absorption, with median time to maximum plasma concentration ranging from 2.0 to 3.9 h across the dose groups (Table S2). After reaching peak plasma concentration, the plasma concentration of mivavotinib declined in a bi‐ or triphasic manner. The terminal half‐life of mivavotinib was too long to be accurately determined from the single‐dose PK data collected over 24 h. Based on dose‐normalized maximum plasma concentration and area under the plasma concentration–time curve from time 0 to 24 h (AUC_24h_), the increase in mivavotinib plasma exposure generally appeared dose‐proportional across the 60–100 mg dose range. Moderate accumulation in exposure was observed, with the geometric mean accumulation ratio of AUC_24h_ ranging from 1.708 to 2.674 across dose groups. The geometric mean peak/trough ratio ranged from 2.975 to 3.891 on cycle 1 day 15.

### 3.7 Efficacy

Thirty‐two patients were response‐evaluable, 17 (70.8%) in dose‐escalation, and 15 (88.2%) in the expansion phase. Response data, including ORR, by dose cohort are shown in Table S3. Among the 17 response‐evaluable patients in the dose‐escalation phase, one (5.9%) patient with breast cancer in the mivavotinib 80 mg dose cohort achieved a confirmed partial response, with a DOR of 4.6 months, and an additional 11 (64.7%) patients had SD, yielding a disease control rate of 70.6%. Of 15 response‐evaluable TNBC patients in the expansion phase, four (26.7%) had SD, which lasted for more than 6 months in two patients. Overall, seven (21.9%) patients remained progression‐free at 6 months. Median PFS was 2.6 months (95% CI: 1.7–3.7 months), and median OS was 6.4 months (95% CI: 3.9, 8.3 months) in the safety population (Table S4).

## 4 DISCUSSION

In preclinical studies, mivavotinib as a single agent or in combination with anti‐PD‐1 antibody reduced the population of immunosuppressive immune cells (Treg cells, MDSCs, and M2 macrophages) and also resulted in complete tumor growth suppression, prolonged tumor‐free survival and potential immune memory against tumor cells in multiple syngeneic or xenograft models. These data suggest that in tumors where SYK‐mediated MDSC immunosuppression is active, mivavotinib can exhibit a therapeutic advantage in combination with a PD‐1 inhibitor. Based on these preclinical results, a phase Ib study of mivavotinib in combination with nivolumab in patients with advanced solid tumors was conducted. During dose‐escalation of combination treatment, one of nine patients receiving 80 mg had a DLT of grade 4 lipase increased and one of four patients dosed at 100 mg QD had a DLT of grade 3 pyrexia. Based on this latter DLT and long‐term tolerability concerns at the 100 mg dose level reported in another study of single‐agent mivavotinib,^28^ the decision was made to focus subsequent enrollment on the lower doses, and the MTD of mivavotinib plus nivolumab was not defined. Additional patients were then enrolled into the mivavotinib 60 and 80 mg QD cohorts to enrich the safety and tolerability data. While both mivavotinib 60 and 80 mg QD in combination with nivolumab showed acceptable safety and tolerability, the RP2D for mivavotinib QD in combination with nivolumab 3 mg/kg Q2W was determined as 80 mg based on a comprehensive evaluation of long‐term safety, tolerability, and preliminary response data. Indeed, one patient with breast cancer had a confirmed partial response at the 80 mg dose level.

Although the study was originally designed with three indications (NSCLC, HNSCC and TNBC), a decision was made by the sponsor to limit enrollment in the expansion phase to patients with metastatic TNBC based on this patient with breast cancer achieving a confirmed response during dose‐escalation. However, the study was terminated early by the sponsor after a review of the TNBC expansion cohort (conducted after ~50% enrollment), indicated that there was insufficient activity to pass an ad hoc futility threshold.

The AE profile of mivavotinib in combination with nivolumab was consistent with those reported previously for single‐agent mivavotinib and single‐agent nivolumab.^12^, ^28^, ^30^ Common TEAEs related to mivavotinib only included pyrexia, amylase increased, and AST increased, while the most frequent TEAE related to nivolumab only was rash; common TEAEs related to both drugs included diarrhea, nausea, AST elevation, and fatigue. Pneumonitis was the most common grade ≥3 TEAE (*n* = 5, 12.2%), and was considered related to both mivavotinib and nivolumab in three patients, and related to nivolumab alone in two patients. Serious TEAEs of pneumonitis resulted in treatment discontinuation in four of 17 patients in the dose‐expansion phase, with pneumonitis thus identified as an overlapping toxicity. Two meta‐analyses of previous studies have reported rates of ~1% for grade ≥3 pneumonitis with anti‐PD‐1 monotherapy across cancer types, with only slightly increased rates for combination therapy.^31^, ^32^ In another meta‐analysis, the risk of pneumonitis was significantly increased with anti‐PD‐1/PD‐L1 therapies in TNBC (odds ratio: 2.52; 95% CI: 1.02–6.26).^33^ The reasons for the higher rates of pneumonitis in this study are unknown, but it is generally agreed that this immune‐related AE is tumor‐specific,^31^, ^32^ occurring due to the immunosuppressive effects of the anti‐PD‐(L)1 drug combination^33^; nevertheless, underlying reasons for this warrant further research. Notwithstanding the rates of pneumonitis, mivavotinib in combination with nivolumab appeared to have a generally manageable safety and tolerability profile.

Single‐agent mivavotinib has previously demonstrated activity in hematologic malignancies, including in phase I/Ib studies in B‐cell lymphoma^28^ and acute myeloid leukemia.^30^ In hematologic malignancies, the antitumor activity of mivavotinib may be the result of direct inhibition of SYK/FLT3 signaling within the malignant cell, whereas in solid tumors, inhibition of SYK/FLT3 by mivavotinib was predicted to reduce cell populations of MDSCs in the TIME to prevent tumor immune escape. The patient population in this study had advanced stage disease, likely involving multiple tumor immune escape mechanisms that may limit the impact of a single agent targeting the TIME. The lack of activity observed in patients with TNBC may also reflect the challenges of the translatability of preclinical data in specific cell models into the clinical setting of various tumor types, or the complexity of the tumor microenvironment in a specific tumor type.

Assessment of paired tumor biopsy samples from three patients by immunohistochemistry showed that treatment with mivavotinib resulted in changes in population numbers of CD8, CD4, Treg, NK, and B cells in the tumor. The changes were, however, not consistent between the three patients; these differences may be attributable to the discontinuation of mivavotinib in one patient and a subsequent rebound in the infiltrating immune cells once mivavotinib had cleared the system prior to the second biopsy, but this observation would need to be confirmed in a larger cohort of patients.

Notably, CD68+ macrophage levels decreased from baseline in all three biopsy pairs, which suggests that treatment with mivavotinib impacts the myeloid lineage within the tumor and may result in the depletion of TAMs. Similar to the observed decrease in TAMs, a decrease in peripheral monocytes following treatment with mivavotinib was also observed suggesting that mivavotinib impacted not only macrophages within the tumor but also monocytes. Inhibition of TAMs is of substantial interest as there is evidence that macrophages adopt a phenotype that promotes tumor growth, angiogenesis, invasion, and metastasis when they enter the tumor microenvironment^34^; consequently, there are numerous TAM inhibitors in clinical development, including inhibitors of CSF1 or CSF1R aimed at sensitizing tumors to the effects of other immunotherapies.^35^ Similar depletion of monocytes was observed in patients treated with trabectedin, but clinical use of trabectedin is limited by its toxicity.^36^, ^37^ Whether mivavotinib impacts the recruitment of new monocytes, differentiation of monocytes into TAMs, or the viability of TAMs is the focus of ongoing investigations. This effect has not been reported previously in studies of other SYK or FLT3 inhibitors and may represent a novel mechanism of action of mivavotinib.

Expression of PD‐L1 appeared to increase in all biopsied samples. PD‐L1 is known to be regulated in response to immune cell activation, and so these data support the idea that mivavotinib is impacting the immune milieu in the tumor. These combined data suggest a rationale for further investigation into the impact of mivavotinib on the tumor microenvironment and the need to identify an optimal combination partner and schedule in which to dose mivavotinib. Our interpretation of these data is limited by the very small sample size and an inability to evaluate the impact of changes in the tumor microenvironment on antitumor activity, as all three patients experienced disease progression soon after the day 15 biopsies were obtained.

Mivavotinib exposure was generally dose proportional with a long terminal half‐life consistent with that of single‐agent mivavotinib in a previous study,^28^ suggesting that the addition of nivolumab to mivavotinib does not notably impact PK parameters, which was consistent with pre‐study predictions of a low risk of drug–drug interactions between mivavotinib and nivolumab.

Overall, mivavotinib in combination with nivolumab in patients with advanced solid tumors had a manageable safety profile, satisfactory PK profile, evidence of a novel pharmacodynamic effect, and preliminary activity. The activity observed, however, was not sufficient to justify further development of this particular combination in patients with metastatic TNBC who have received at least one prior line of chemotherapy. Previous studies have demonstrated potential for diminished responses to immunotherapies in patients with TNBC who have received prior chemotherapy,^38^ which could have contributed to the low response rate in this study. Instead, chemotherapy‐based combinations may be needed in this setting; for example, the non‐comparative phase II TONIC trial of nivolumab after induction treatment including irradiation, cyclophosphamide, cisplatin, or doxorubicin, in patients with metastatic TNBC demonstrated an ORR of 20%.^39^ This is also the case for other immuno‐oncology combinations in TNBC: low rates of activity have been observed with single‐agent pembrolizumab,^38^ and it is only approved in combination with chemotherapy in TNBC.^40^ Additionally, IPI‐549, which targets tumor‐associated myeloid cells through selective inhibition of PI3K‐gamma, demonstrated promising efficacy when administered as a triplet therapy with both atezolizumab and chemotherapy in first‐line.^41^ In contrast, here we investigated mivavotinib as a doublet with nivolumab in patients who had received at least one prior therapy. It is also important to continue to evaluate the safety and antitumor activity of mivavotinib combination therapy in other solid cancer types associated with MDSC‐mediated tumor immunosuppression, including colon/colorectal cancer, a tumor type shown to respond to mivavotinib plus anti‐PD1 in a preclinical model.^5^, ^18^ Notably, findings from a recently completed phase I study (NCT03756818) of mivavotinib combined with paclitaxel in patients with advanced solid tumors, including high‐grade epithelial ovarian cancer, are highly anticipated.

## 5 CONCLUSIONS

The safety profile of mivavotinib in combination with nivolumab in patients with advanced solid tumors was notable for a higher‐than‐expected rate of pneumonitis. Mivavotinib was rapidly absorbed, and plasma exposure was dose‐proportional. Mivavotinib plus nivolumab did not demonstrate sufficient antitumor activity in patients with relapsed/refractory solid tumors, but a novel pharmacodynamic effect was seen that was not anticipated based on the primary mechanism of action of mivavotinib.

## AUTHOR CONTRIBUTIONS

**Dejan Juric:** Conceptualization (lead); investigation (lead); writing – original draft (lead); writing – review and editing (lead). **Minal Barve:** Investigation (equal); project administration (equal); writing – review and editing (equal). **Ulka Vaishampayan:** Investigation (equal); methodology (equal); project administration (equal); writing – review and editing (equal). **Desamparados Roda:** Investigation (equal); methodology (equal); project administration (equal); writing – original draft (equal); writing – review and editing (equal). **Aitana Calvo:** Investigation (equal); methodology (equal); supervision (equal); validation (equal); writing – review and editing (equal). **Noelia Martinez Jañez:** Conceptualization (equal); supervision (equal); writing – review and editing (equal). **Jose Trigo:** Data curation (equal); investigation (equal); resources (equal); writing – review and editing (equal). **Alastair Greystoke:** Investigation (equal); resources (equal); writing – review and editing (equal). **R. Donald Harvey:** Investigation (equal); resources (equal); supervision (equal); writing – original draft (equal); writing – review and editing (equal). **Anthony J. Olszanski:** Data curation (equal); investigation (equal); writing – review and editing (equal). **Mateusz Opyrchal:** Investigation (equal); writing – review and editing (equal). **Alexander Spira:** Conceptualization (equal); data curation (equal); formal analysis (equal); funding acquisition (equal); investigation (equal); resources (equal); supervision (equal); validation (equal); writing – review and editing (equal). **Fiona Thistlethwaite:** Data curation (equal); investigation (equal); writing – original draft (equal); writing – review and editing (equal). **Begoña Jiménez:** Investigation (equal); supervision (equal); writing – review and editing (equal). **Jessica Huck Sappal:** Data curation (equal); conduction of experiments, provision of data for Figure 1; writing – review and editing (equal). **Karuppiah Kannan:** Conceptualization (equal); project administration (equal); resources (equal); writing – review and editing (equal). **Jason Riley:** Investigation (equal); validation (equal); writing – review and editing (equal). **Cheryl Li:** Formal analysis (equal); writing – review and editing (equal). **Cong Li:** Formal analysis (equal); writing – review and editing (equal). **Richard C. Gregory:** Data curation (equal); formal analysis (equal); investigation (equal); visualization (equal); writing – original draft (equal); writing – review and editing (equal). **Harry Miao:** Conceptualization (equal); data curation (equal); formal analysis (equal); resources (equal); supervision (equal); writing – review and editing (equal). **Shining Wang:** Data curation (equal); formal analysis (equal); methodology (equal); writing – review and editing (equal).

## CONFLICT OF INTEREST STATEMENT

Dejan Juric reports consulting fees from Novartis, Genentech, Syros, Eisai, Vibliome, PIC Therapeutics, Mapkure, Relay Therapeutics, and Eli Lilly; and grants or funds from Novartis, Genentech, Syros, Eisai, Pfizer, Amgen, InventisBio, Arvinas, Takeda, Blueprint, AstraZeneca, Ribon Therapeutics, Infinity, and Eli Lilly. Ulka Vaishampayan reports honoraria from Sanofi, Bayer, and Exelixis; consulting fees from BMS, Bayer, Alkermes, Gilead, Exelixis, Merck and Pfizer; and grants or funds from BMS and Exelixis. Desamparados Roda reports an advisory council or committee position for Abbvie M19‐345 Phase 1 program. Jose Trigo reports advisory council or committee positions at AstraZeneca, BMS, Bayer, EISAI, MSD, and Janssen; and grants or funds from MSD, BMS and AstraZeneca. Alastair Greystoke reports honoraria and consulting fees from Takeda. R. Donald Harvey reports consulting fees from Amgen and GlaxoSmithKline; and research funding to their institution that supports their salary from Abbisko, AbbVie, Actuate, Amgen, AstraZeneca, Bayer, Bristol‐Myers Squibb, Boston Biomedical, Genmab, GlaxoSmithKline, Infinity, InhibRx, Janssen, Merck, Mersana, Meryx, Morphosys, Nektar, Novartis, Pfizer, Regeneron, Sanofi, Sutro, Takeda, Turning Point Therapeutics, and Xencor. Anthony J. Olszanski reports advisory council or committee positions for Merck, BMS, Novartis, Eisai, Nektar, and InstilBio; and honoraria from Pfizer. Mateusz Opyrchal reports advisory council or committee position for Alphageneron; and grants or funds from Eli Lilly and Pfizer. Alexander Spira reports employment at NEXT Oncology Virginia; ownership of stocks/shares at Eli Lilly; honoraria from CytomX Therapeutics, AstraZeneca/MedImmune, Merck, Takeda, Amgen, Janssen Oncology, Novartis, Bristol‐Myers Squibb, and Bayer; consulting fees from Incyte, Amgen, Novartis, Mirati Therapeutics, Gristone Oncology, Jazz Pharmaceuticals, Takeda, Janssen, Mersana, Daiichi Sankyo/AstraZeneca, Regeneron, Lilly and Black Diamond Therapeutics; and grants or funds from LAM Therapeutics, and Regeneron. Fiona Thistlethwaite reports advisory council or committee position at T‐knife Therapeutics (scientific advisory board member); honoraria from Kite; ad hoc consultancy fees from Adicet Bio, BMS, F‐Star, GSK, Ixaka, Janssen, Leucid, and QPCTL Scenic Biotech in the last 2 years; and grants or funds from GSK. Begoña Jiménez reports speaker fees from Roche, Daichii Sankyo, and Lilly; and travel funds from Gilead. Karuppiah Kannan and Jason Riley report employment with Takeda. Cheryl Li, Cong Li, Richard C. Gregory, Harry Miao, and Shining Wang report employment and ownership of stocks/shares with Takeda. Minal Barve, Aitana Calvo, Noelia Martinez Jañez, and Jessica Huck Sappal declare no potential conflicts of interest.

## FUNDING INFORMATION

This study was funded by Takeda Development Center Americas, Inc. (TDCA), Lexington, MA, USA.

## ETHICS STATEMENT

The study was conducted in accordance with the International Council for Harmonisation Good Clinical Practice standards, the Declaration of Helsinki, and all applicable regulatory requirements. Relevant institutional review boards or ethics committees approved all aspects of the study, and all authors had access to primary clinical trial data. All patients provided written informed consent. This trial is registered at ClinicalTrials.gov Identifier: NCT02834247 https://clinicaltrials.gov/ct2/show/NCT02834247.

## Supporting information


Data S1.

## Data Availability

Requests for de‐identified datasets for the results reported in this publication will be made available to qualified researchers following submission of a methodologically sound proposal. Data will be made available for such requests following online publication of this article and for 1 year thereafter in compliance with applicable privacy laws, data protection, and requirements for consent and anonymization. Calithera does not share identified participant data or a data dictionary.
