# Supplementary material for: A phase Ib study evaluating the recommended phase II dose, safety, tolerability, and efficacy of mivavotinib in combination with nivolumab in advanced solid tumors
Source: Cancer Med. 2024 Mar 19;13(5):10.1002/cam4.6776. doi: 10.1002/cam4.6776 (PMC10949085; doi:10.1002/cam4.6776)
Supplement: Supplementary file 1 — Data S1. [file CAM4-13--s001.docx]

**Supplementary Material**

**Title:** A phase Ib study evaluating the recommended phase II dose, safety, tolerability, and efficacy of mivavotinib in combination with nivolumab in advanced solid tumors

**Authors:** Dejan Juric^1^ | Minal Barve^2^ | Ulka Vaishampayan^3^ | Desamparados Roda^4^ | Aitana Calvo^5^ | Noelia Martinez Jañez^6^ | Jose Trigo^7^ | Alastair Greystoke^8^ | R. Donald Harvey^9^ | Anthony J. Olszanski^10^ | Mateusz Opyrchal^11^ | Alexander Spira^12,13^ | Fiona Thistlethwaite^14^ | Begoña Jiménez^7^ | Jessica Huck Sappal^15^ | Karuppiah Kannan^16^ | Jason Riley^17^ | Cheryl Li^18^ | Cong Li^19^ | Richard C. Gregory^15^ | Harry Miao^20^ | Shining Wang^21^

**Affiliations:**
^1^Termeer Center for Targeted Therapies, Massachusetts General Hospital Cancer Center, Boston, Massachusetts, USA
^2^Medical Oncology, Mary Crowley Cancer Research, Dallas, Texas, USA
^3^Internal Medicine/Oncology, Karmanos Cancer Institute, Wayne State University, Detroit, Michigan, USA
^4^Department of Medical Oncology, University Hospital, Valencia, Spain
^5^Medical Oncology, Instituto de Investigación Sanitaria Gregorio Marañón, Madrid, Spain
^6^Department of Oncology, Hospital Universitario Ramón y Cajal, Madrid, Spain
^7^Medical Oncology, Hospital Universitario Virgen de la Victoria, Málaga, Spain
^8^Faculty of Medical Sciences, Newcastle University, Newcastle upon Tyne, UK
^9^Hematology and Medical Oncology, Winship Cancer Institute of Emory University, Atlanta, Georgia, USA
^10^Department of Hematology/Oncology, Fox Chase Cancer Center, Philadelphia, Pennsylvania, USA
^11^Division of Oncology, Washington University School of Medicine in St Louis, St Louis, Missouri, USA
^12^Medical Oncology, Johns Hopkins School of Medicine, Johns Hopkins University, Baltimore, Maryland, USA
^13^Medical Oncology, Virginia Cancer Specialists, US Oncology Research, NEXT Oncology Virginia, Leesburg, Virginia, USA
^14^Medical Oncology, The Christie NHS Foundation Trust and University of Manchester, Manchester, UK
^15^Precision and Translational Medicine, Takeda Development Center Americas, Inc. (TDCA), Lexington, Massachusetts, USA
^16^Oncology Therapeutic Area Unit, Takeda Development Center Americas, Inc. (TDCA), Lexington, Massachusetts, USA
^17^Gastroenterology, Takeda Development Center Americas, Inc. (TDCA), Lexington, Massachusetts, USA
^18^Quantitative Clinical Pharmacology, Takeda Development Center Americas, Inc. (TDCA), Lexington, Massachusetts, USA
^19^Statistical and Quantitative Sciences, Takeda Development Center Americas, Inc. (TDCA), Lexington, Massachusetts, USA
^20^Clinical Development, Takeda Development Center Americas, Inc. (TDCA), Lexington, Massachusetts, USA
^21^Takeda Oncology Clinical Science, Takeda Development Center Americas, Inc. (TDCA), Lexington, Massachusetts, USA

**Corresponding Author:** Dejan Juric, MD, Massachusetts General Hospital Cancer Center, 55 Fruit Street, Boston, MA 02114, USA. Tel: 617-726-6500; Fax: 617-724-1079; Email: juric.dejan@mgh.harvard.edu

**Supplementary Methods**

**Definition of Dose-Limiting Toxicity**

A dose-limiting toxicity (DLT) was defined as any of the following events that were considered by the investigator to be at least possibly related to therapy with study drug, either mivavotinib or nivolumab or both (adverse events in which the relationship to study drug could not be ruled out were considered possibly related to study drug):

- Grade 4 neutropenia [absolute neutrophil count (ANC) <500 cells/mm^3^] unresolved to ≤grade 1 (ANC >1500 cells/mm^3^) or baseline for >7 consecutive days in the absence of growth factor support.
- Grade 3 neutropenia (ANC <1000 cells/mm^3^) with fever and/or infection, where fever was defined as an oral temperature ≥38.5 ºC.
- Grade 4 thrombocytopenia (<25,000 platelets/mm^3^) unresolved to ≤grade 1 (>75,000 platelets/mm^3^) or baseline for >7 consecutive days or a platelet count <10,000/mm^3^ at any time.
- Grade ≥3 thrombocytopenia (<50,000 platelets/mm^3^) with clinically significant bleeding.
- Grade 4 anemia.
- Any grade ≥3 nonhematological toxicity with the following exceptions:
  - Grade ≥3 nausea and/or vomiting that had not resolved to <grade 3 within 72 hours upon optimal antiemetic and/or antidiarrheal treatment. All patients were to receive optimal antiemetic and/or antidiarrheal treatment according to standard of care (SOC). An optimal antiemetic regimen was defined as one that employs both a 5-hydroxytryptamine 3 serotonin receptor antagonist and a corticosteroid given in standard doses and according to standard schedules.
  - Transient grade 3 fatigue (≤1 week).
  - Grade 3 arthralgia/myalgia.
  - Asymptomatic lipase elevation (<grade 4) in the absence of significant amylase elevation (<grade 3) considered not dose limiting following agreement between the sponsor and investigators.
  - Asymptomatic amylase elevation (<grade 4) in the absence of significant lipase elevation (<grade 3) considered not dose limiting following agreement between the sponsor and investigators.
  - Asymptomatic grade 3 elevation of a single liver enzyme (aspartate aminotransferase or alanine aminotransferase) in the absence of significant bilirubin elevation (<grade 3) considered not dose limiting following agreement between the sponsor and investigators.
  - Isolated ≥grade 3 abnormalities of other laboratory parameters that resolved to ≤grade 1 in ≤7 days without clinical sequelae or need for therapeutic intervention considered not dose limiting following agreement between the sponsor and investigators.
  - Grade 3 rash lasting ≤7 days with optimal treatment that includes topical steroid treatment, oral antihistamines, and pulse oral steroids, if necessary.

**Full Inclusion Criteria**

Each patient was required to meet all the following inclusion criteria to be enrolled in the study:

1. Male or female patients aged 18 years or older.
2. Eastern Cooperative Oncology Group performance status (ECOG PS) 0 or 1.
3. Female patients who:
   1. Were postmenopausal for at least 1 year before the Screening visit, OR
   2. Were surgically sterile, OR
   3. If they were of childbearing potential, agreed to practice one highly effective method of contraception and one additional effective (barrier) method, at the same time, from the time of signing the informed consent through 180 days after the last dose of study drug, OR
   4. Agreed to practice true abstinence, when this is in line with the preferred and usual lifestyle of the patient. [Periodic abstinence (e.g., calendar, ovulation, symptothermal, post-ovulation methods) withdrawal, spermicides only, and lactational amenorrhea were not acceptable methods of contraception. Female and male condoms were not to be used together].
4. Male patients, even if surgically sterilized (i.e., status post vasectomy), who:
   1. Agreed to practice effective barrier contraception during the entire study treatment period and through 180 days after the last dose of study drug, OR
   2. Agreed to practice true abstinence, when this is in line with the preferred and usual lifestyle of the patient. [Periodic abstinence (e.g., calendar, ovulation, symptothermal, post-ovulation methods) withdrawal, spermicides only, and lactational amenorrhea were not acceptable methods of contraception. Female and male condoms were not to be used together].
5. Voluntary written consent was required before performance of any study-related procedure not part of standard medical care, with the understanding that consent may be withdrawn by the patient at any time without prejudice to future medical care.
6. Suitable venous access for the study-required blood sampling, including pharmacokinetic (PK) and pharmacodynamic sampling.
7. Clinical laboratory values and other measures as specified below within 28 days before the first dose of study drug:
   1. Total bilirubin was required to be ≤1.5×the upper limit of normal (ULN).
   2. Alanine aminotransferase and aspartate aminotransferase were required to be ≤2.5×ULN.
   3. Creatinine clearance was required to be ≥60 mL/minute as estimated by the Cockcroft-Gault equation or based on urine collection (12 or 24 hours).
   4. Hemoglobin was required to be ≥9 g/dL, absolute neutrophil count was required to be ≥1500/µL, and platelet count was required to be ≥75,000/µL.
   5. Lipase was required to be ≤1.5×ULN and amylase ≤1.5×ULN with no clinical symptoms suggestive of pancreatitis and cholecystitis.
   6. Blood pressure ≤grade 1 (hypertensive patients were permitted if their blood pressure was controlled to ≤grade 1 by hypotensive medications and glycosylated HbA1C ≤6.5%).
8. Recovered (i.e., ≤grade 1 toxicity) from the reversible effects of prior anticancer therapy.
9. To be enrolled in the dose escalation phase of the study, patients were required to have:
   1. A histologically confirmed diagnosis of advanced solid tumor with a radiographically or clinically evaluable disease. Measurable disease as defined by Response Evaluation Criteria in Solid Tumors (RECIST) version 1.1, was not required for participation in the dose-escalation phase of the study.
   2. One or more prior lines of therapy and no effective therapeutic options available based on investigator assessment. Prior exposure to marketed immune checkpoint inhibitors, such as nivolumab and pembrolizumab, was permitted during dose escalation.
10. To be enrolled in the triple-negative breast cancer (TNBC) expansion cohort, patients were required to have:
    1. Histologically confirmed, metastatic TNBC with measurable disease per Response Evaluation Criteria in Solid Tumours RECIST version 1.1.
    2. Triple-negative disease (estrogen receptor, progesterone receptor, and HER2 negativity) confirmed on a histological biopsy of a metastatic tumor lesion (receptor conversion not allowed).
    3. Safely accessible tumor lesions (based on investigator’s assessment) for serial pre-treatment and post-treatment biopsies were required for patients receiving mivavotinib monotherapy run-in treatment for two weeks followed by mivavotinib plus nivolumab combination treatment (~10/30 response-evaluable patients); adequate, newly obtained, core or excisional biopsy of a metastatic tumor lesion not previously irradiated was required. Mandatory biopsies were taken before mivavotinib monotherapy, after the two weeks of mivavotinib monotherapy, and after six weeks of mivavotinib plus nivolumab combination therapy. An optional biopsy was permitted to be taken disease progression with additional consent from the patient.
    4. One, two, or three prior lines of chemotherapy for metastatic disease and with progression of disease on last treatment regimen.
    5. For the purposes of this study, neoadjuvant and/or adjuvant chemotherapy regimens did not count as a prior line of therapy.
    6. Prior treatment must include an anthracycline and/or a taxane in the neoadjuvant, adjuvant, or metastatic setting with the exception for patients who were clinically contraindicated for these chemotherapies.

**Full Exclusion Criteria**

Patients meeting any of the following exclusion criteria were not to be enrolled in the study:

1. Active brain metastases or leptomeningeal metastases.
2. Active or suspected autoimmune disease or a history of known autoimmune disease, with the exception of patients with vitiligo, type I diabetes mellitus, resolved childhood asthma/atopy, residual hypothyroidism due to an autoimmune condition requiring only hormone replacement, psoriasis not requiring systemic treatment, or conditions not expected to recur in the absence of an external trigger.
3. Any condition requiring systemic treatment with corticosteroids (>10 mg daily prednisone equivalents) or other immunosuppressive medications within 14 days before first dose of study drug. Corticosteroids for topical use or in nasal spray were allowed, as were inhaled steroids and adrenal replacement steroid doses >10 mg daily in the absence of active autoimmune disease.
4. History of pneumonitis requiring treatment with steroids; history of idiopathic pulmonary fibrosis, drug-induced pneumonitis, organizing pneumonia, or evidence of active pneumonitis on the screening chest computerized tomography scan; history of radiation pneumonitis in the radiation field (fibrosis) was permitted.
5. History of interstitial lung disease.
6. Prior therapy with experimental antitumor vaccines; any T-cell co-stimulation agents or inhibitors of checkpoint pathways, such as anti-programmed death-1 receptor (PD-1), anti-programmed death-ligand 1 (PD-L1), anti-PD-L2, anti-CD137, or anti-CTLA-4 antibody; or other agents specifically targeting T-cells were prohibited. However, for dose escalation, prior treatment with the marketed inhibitors of the immune checkpoint pathway, such as nivolumab and pembrolizumab, was allowed. In addition, in each of the expansion cohorts, six response-evaluable patients with prior exposure to anti-PD-1 or anti-PD-L1 agents (including both marketed and investigational) could enroll.
7. Any serious medical or psychiatric illness, including drug or alcohol abuse, that could have, in the investigator’s opinion, potentially interfered with the completion of treatment according to the study protocol.
8. Life-threatening illness unrelated to cancer.
9. Female patients who were lactating and breast-feeding or a positive serum pregnancy test during the screening period or a positive urine pregnancy test on Day 1 before the first dose of study drug.
10. Systemic anticancer treatment (including investigational agents) or radiotherapy <2 weeks before the first dose of study treatment (≤4 weeks for antibody-based therapy including unconjugated antibody, antibody-drug conjugate, and bi-specific T-cell engager agents; ≤8 weeks for cell-based therapy or antitumor vaccine) or had not recovered from acute toxic effects from prior chemotherapy and radiotherapy.
11. Prior treatment with investigational agents ≤21 days (≤4 weeks for monoclonal antibodies with evidence of PD) or ≤5×their half-lives (whichever is shorter) before the first dose of study treatment. A minimum of 10 days was to elapse from prior therapy to initiating protocol therapy.
12. Major surgery within 14 days before the first dose of study drug and not recovered fully from any complications from surgery.
13. Systemic infection requiring intravenous antibiotic therapy or other serious infection within 14 days before the first dose of study drug.
14. Known human immunodeficiency virus positive (testing not required).
15. Known hepatitis B surface antigen-positive or known or suspected active hepatitis C infection (testing not required).
16. Patients with another malignancy within two years of study start. Patients with nonmelanoma skin cancer or carcinoma in situ of any type were not excluded if they had undergone complete resection and were considered disease-free at the time of study entry.
17. Any clinically significant comorbidities, such as uncontrolled pulmonary disease, known impaired cardiac function or clinically significant cardiac disease (specified below), active central nervous system disease, active infection, or any other condition that could compromise the patient’s participation in the study. Patients with any of the following cardiovascular conditions were excluded:
    1. Acute myocardial infarction within six months before starting study drug.
    2. Current or history of New York Heart Association Class III or IV heart failure.
    3. Evidence of current uncontrolled cardiovascular conditions including cardiac arrhythmias, angina, pulmonary hypertension, or electrocardiographic evidence of acute ischemia or active conduction system abnormalities.
    4. Fridericia corrected QT interval >450 milliseconds (men) or >475 milliseconds (women) on a 12-lead electrocardiogram (ECG) during the screening period.
    5. Abnormalities on 12-lead ECG including, but not limited to, changes in rhythm and intervals that in the opinion of the investigator were considered to be clinically significant.
18. Known gastrointestinal disease or gastrointestinal procedure that could interfere with the oral absorption or tolerance of mivavotinib including difficulty swallowing tablets; diarrhea grade >1 despite supportive therapy.
19. Use or consumption of any of the following substances:
    1. Medications or supplements that are known to be inhibitors of P-glycoprotein (P-gp) and/or strong reversible inhibitors of CYP3A within five times the inhibitor half-life (if a reasonable half-life estimate was known) or within seven days (if a reasonable half-life estimate was unknown) before the first dose of study drug. In general, the use of these agents was not permitted during the study.
    2. Medications or supplements that are known to be strong CYP3A mechanism-based inhibitors or strong CYP3A inducers and/or P-gp inducers within seven days, or within five times the inhibitor or inducer half-life (whichever was longer), before the first dose of study drug. In general, the use of these agents was not permitted during the study.
    3. Non-oncology vaccine therapies for prevention of infectious diseases [e.g., human papillomavirus vaccine] within four weeks of study drug administration. It was permitted that the inactivated seasonal influenza vaccine could be given to patients before treatment and while on therapy without restriction. Influenza vaccines containing live virus or other clinically indicated vaccinations for infectious diseases (e.g., pneumovax, varicella) was possibly permitted but a discussion was required first with the sponsor’s medical monitor and a washout period may have been required before and after administration of vaccine.
    4. Grapefruit-containing food or beverages within five days before the first dose of study drug. Note that grapefruit-containing food and beverages were prohibited during the study.
20. For dose-expansion patients who had tumor biopsies collected:
    1. ECOG PS >1.
    2. Activated partial thromboplastin time or plasma thromboplastin outside the institution’s SOC.
    3. Platelet count <75,000/µL.
    4. Known bleeding diathesis or history of abnormal bleeding, or any other known.
    5. Coagulation abnormalities that would contraindicate the tumor biopsy procedure.
    6. Ongoing therapy with any anticoagulant or antiplatelet agents (e.g., aspirin, clopidogrel, coumadin, heparin, or warfarin) that could not be held to permit tumor biopsy.

**Additional Pharmacodynamics Methods**

*Immunohistochemistry Staining*

Staining was performed using a single 4 µM formalin-fixed paraffin-embedded slide. For each staining round, two cyanine dye-labeled (Cy3, Cy5) antibodies were paired and used to highlight two markers; the staining signal was then imaged on multiple regions of interest within the tumor biopsies. Subsequently, novel dye inactivation was used to enable repeated rounds of staining. Proprietary deep learning-based workflows were applied to identify individual cells and to perform cell classification based on the expression of all individual markers based in the selected regions of interest.

*Flow Cytometry Cell Markers*

| **T-cells** | **B-cells** | **NK and dendritic cells** | **MDSCs and monocytes** |
| --- | --- | --- | --- |
| CD45 | CD45 | CD45 | CD45 |
| CD3 | CD19 | CD3 | CD3/CD19/CD56 dump |
| CD4 | CD3 | CD19 | CD14 |
| CD8 | CD27 | CD16 | HLA-Dr |
| CD25 | CD24 | CD56 | CD16 |
| CD127 | CD20 | CD14 | CD15 |
| CD45RA | CD38 | HLA-DR | CD66b |
| CCR7 | IgM | CD11c | CD11b |
| CD28 | IgG | CD123 | CD33 |
| PD-1 | IgD |  |  |

HLA-DR, human leukocyte antigen – DR isotype; IgD, immunoglobulin D; IgG, immunoglobulin G; IgM, immunoglobulin M; MDSCs, myeloid-derived suppressor cells; NK, natural killer; PD-1, programmed death-1 receptor.

**Study Populations**

The DLT-evaluable population included patients who experienced a DLT during cycle 1 and/or patients who completed ≥75% of the planned doses of mivavotinib plus two doses of nivolumab in cycle 1 and who had sufficient follow-up data to determine whether a DLT had occurred. The response-evaluable population comprised patients who received ≥1 dose of either study drug, had measurable disease at baseline, and had ≥1 postbaseline disease assessment. The safety population included all enrolled patients who received ≥1 dose of either study drug. The PK-evaluable population included all patients in the dose-escalation phase with sufficient plasma concentration-time and dosing data to reliably estimate mivavotinib PK parameters.

**Supplementary Table S1.** Overall summary of incidence rates of treatment-emergent adverse events.

|  | **Dose escalation** | | | | **Expansion** |  |
| --- | --- | --- | --- | --- | --- | --- |
|  | **Mivavotinib**  **60 mg QD (n = 8)** | **Mivavotinib 80 mg QD (n = 11)** | **Mivavotinib 100 mg QD (n = 5)** | **Total**  **(N = 24)** | **Mivavotinib**  **80 mg QD**  **(n = 17)** | **Total (N = 41)** |
| Any TEAE, n *(%)* | 8 (100) | 11 (100) | 5 (100) | 24 (100) | 17 (100) | 41 (100) |
| Any mivavotinib-related TEAE | 6 (75.0) | 9 (81.8) | 5 (100) | 20 (83.3) | 7 (41.2) | 27 (65.9) |
| Any nivolumab-related TEAE | 5 (62.5) | 3 (27.3) | 4 (80.0) | 12 (50.0) | 4 (23.5) | 16 (39.0) |
| Any TEAE related to both mivavotinib and nivolumab | 6 (75.0) | 4 (36.4) | 2 (40.0) | 12 (50.0) | 9 (52.9) | 21 (51.2) |
| Any grade ≥3 TEAE, n (%) | 7 (87.5) | 9 (81.8) | 5 (100) | 21 (87.5) | 13 (76.5) | 34 (82.9) |
| Any mivavotinib-related grade ≥3 TEAE | 3 (37.5) | 5 (45.5) | 3 (60.0) | 11 (45.8) | 2 (11.8) | 13 (31.7) |
| Any nivolumab-related grade ≥3 TEAE | 1 (12.5) | 1 (9.1) | 2 (40.0) | 4 (16.7) | 2 (11.8) | 6 (14.6) |
| Any grade ≥3 TEAE related to both mivavotinib and nivolumab | 4 (50.0) | 2 (18.2) | 2 (40.0) | 8 (33.3) | 5 (29.4) | 13 (31.7) |
| Any TEAE leading to discontinuation of: n (%) |  |  |  |  |  |  |
| Mivavotinib only | 0 | 0 | 1 (20.0) | 1 (4.2) | 0 | 1 (2.4) |
| Nivolumab only | 0 | 0 | 1 (20.0) | 1 (4.2) | 2 (11.8) | 3 (7.3) |
| Both mivavotinib and nivolumab | 2 (25.0) | 3 (27.3) | 1 (20.0) | 6 (25.0) | 4 (23.5) | 10 (24.4) |
| Any TEAE leading to dose-interruption of: n (%) |  |  |  |  |  |  |
| Mivavotinib only | 6 (75.0) | 8 (72.7) | 3 (60.0) | 17 (70.8) | 7 (41.2) | 24 (58.5) |
| Nivolumab only | 2 (25.0) | 0 | 1 (20.0) | 3 (12.5) | 1 (5.9) | 4 (9.8) |
| Both mivavotinib and nivolumab | 3 (37.5) | 7 (63.6) | 2 (40.0) | 12 (50.0) | 6 (35.3) | 18 (43.9) |
| Any TEAE leading to dose delay of: n (%) |  |  |  |  |  |  |
| Mivavotinib only | 0 | 1 (9.1) | 0 | 1 (4.2) | 0 | 1 (2.4) |
| Nivolumab only | 2 (25.0) | 1 (9.1) | 1 (20.0) | 4 (16.7) | 1 (5.9) | 5 (12.2) |
| Both mivavotinib and nivolumab | 0 | 1 (9.1) | 0 | 1 (4.2) | 1 (5.9) | 2 (4.9) |
| Any serious TEAE, n (%) | 6 (75.0) | 7 (63.6) | 4 (80.0) | 17 (70.8) | 12 (70.6) | 29 (70.7) |
| Any mivavotinib-related serious TEAE | 1 (12.5) | 0 | 2 (40.0) | 3 (12.5) | 0 | 3 (7.3) |
| Any nivolumab-related serious TEAE | 0 | 0 | 2 (40.0) | 2 (8.3) | 1 (5.9) | 3 (7.3) |
| Any serious TEAE related to both mivavotinib and nivolumab | 1 (12.5) | 1 (9.1) | 1 (20.0) | 3 (12.5) | 3 (17.6) | 6 (14.6) |

Abbreviations: MedDRA, Medical Dictionary for Regulatory Activities; QD, once daily; TEAE, treatment-related adverse event.

MedDRA version 22.0 was used for coding TEAEs. TEAEs were defined as any adverse events that occurred after the administration of the first dose of study drug and through 28 days after the last dose of study treatment or until the start of subsequent anticancer therapy. TEAEs in which the relationship to either mivavotinib or nivolumab could not be ruled out by the investigator were considered treatment-related. In the expansion group, 12 patients received mivavotinib and nivolumab from day 1, and five patients received only mivavotinib on days 1–14, plus nivolumab from Day 15. A dose interruption was a missed dose within a cycle (or a pause during the infusion period for nivolumab) and a dose delay was a postponement of the start of the next cycle.

**Supplementary Table S2.** Mivavotinib plasma pharmacokinetic parameters after single (Day 1) and multiple (Day 15) administration of mivavotinib (60, 80, or 100 mg QD) and nivolumab (3 mg/kg intravenously every two weeks) in cycle 1.

|  | **Day 1** | | | **Day 15** | | |
| --- | --- | --- | --- | --- | --- | --- |
| **Parameter** | **Mivavotinib**  **60 mg QD**  **(n = 8)** | **Mivavotinib**  **80 mg QD**  **(n = 11)** | **Mivavotinib**  **100 mg QD**  **(n = 5)** | **Mivavotinib**  **60 mg QD**  **(n = 5)** | **Mivavotinib**  **80 mg QD**  **(n = 8)** | **Mivavotinib**  **100 mg QD**  **(n = 3)** |
| **Median T_max,_ hours (range)** | 2.2 (0.4–7.5) | 2.1 (0.4–4.1) | 3.7 (0.9–4.1) | 2.0 (1.1–4.0) | 3.9 (2.0–5.0) | 3.8 (2.1–4.0) |
| **Geometric mean C_max_, ng/mL (%CV)** | 84.0 (56.1) | 212.5 (64.7) | 165.6 (55.5) | 132.0 (68.0) | 236.1 (59.0) | 379.8 (26.7) |
| **Geometric mean AUC_24h_, h·ng/mL (%CV)** | 845.7 (24.6) | 1867 (59.5) | 1745 (56.4) | 1906^a^ (28.4) | 2260^b^ (35.4) | 4515 (37.6) |
| **Geometric mean AUC_24h_/dose, h·ng/mL/mg** | 14.1 (24.6) | 23.34^b^ (59.5) | 17.45 (56.4) | 31.77^a^ (28.4) | 28.24^b^ (35.4) | 45.15 (37.6) |
| **Geometric mean CLss/F, L/h (%CV)** | - | - | - | 31.48^a^ (28.4) | 35.41^b^ (35.4) | 22.15 (37.6) |
| **Geometric mean PTR (%CV)** | - | - | - | 2.975 (40.9) | 3.499 (38.2) | 3.891 (16.7) |
| **Geometric mean R_ac_ (AUC_24h_) (%CV)** | - | - | - | 2.674^a^ (26.7) | 1.708^b^ (28.6) | 2.093 (10.8) |
| **Geometric mean R_ac,_ (C_max_) (%CV)** | - | - | - | 1.623 (100.2) | 1.333 (77.0) | 1.728 (16.1) |

Abbreviations: AUC_24h_, area under the plasma concentration versus time curve from time 0–24 hours postdose; C_max_, observed maximum (peak) plasma concentration; CV, coefficient of variation of geometric mean; CLss, calculated steady-state clearance; PK, pharmacokinetics; PTR, peak/trough ratio; QD, once daily; R_ac_, accumulation ratio; T_max_, time to reach observed maximum (peak) plasma concentration.

^a^n = 4 ^b^n = 6.

**Supplementary Table S3.** Summary of overall response rate, disease control rate and progressive disease in the response-evaluable population.

|  | **Dose escalation** | | | | **Expansion** |  |
| --- | --- | --- | --- | --- | --- | --- |
| **Response category** | **Mivavotinib**  **60 mg QD**  **(n = 4)** | **Mivavotinib  80 mg QD  (n = 8)** | **Mivavotinib**  **100 mg QD**  **(n = 5)** | **Total (N = 17)** | **Mivavotinib 80 mg**  **QD (n = 15)** | **Total (N = 32)** |
| **ORR, n (%)** | 0 | 1 (12.5) | 0 | 1 (5.9) | 0 | 1 (3.1) |
| PR | 0 | 1 (12.5) | 0 | 1 (5.9) | 0 | 1 (3.1) |
| SD | 2 (50.0) | 5 (62.5) | 4 (80.0) | 11 (64.7) | 4 (26.7) | 15 (46.9) |
| PD | 2 (50.0) | 2 (25.0) | 1 (20.0) | 5 (29.4) | 11 (73.3) | 16 (50.0) |
| **Disease control rate** |  |  |  |  |  |  |
| CR+PR+SD | 2 (50) | 6 (75.0) | 4 (80.0) | 12 (70.6) | 4 (26.7) | 16 (50.0) |
| **PD rate at 6 months** | 4 (100) | 4 (50.0) | 4 (80.0) | 12 (70.6) | 13 (86.7) | 25 (78.1) |

Abbreviations: CR, complete response; ORR, overall response rate; PD, progressive disease; PR, partial response; QD: once daily; SD, stable disease.

**Supplementary Table S4.** Median progression-free survival and overall survival in the safety population.

|  | **Dose escalation**  **(n *=* 24)** | **Expansion**  **(n *=* 17)** | **Overall**  **(N = 41)** |
| --- | --- | --- | --- |
| **Median PFS (95% CI)** | 3.5 (1.8–5.0) | 1.7 (1.0–2.8) | 2.6 (1.7–3.7) |
| N patients with PD (%) | 19 (79.2) | 15 (88.2) | 34 (82.9) |
| Deaths (%) | 16 (66.7) | 8 (47.1) | 24 (58.5) |
| **Median OS (95% CI)** | 7.2 (3.9–10.7) | 4.9 (3.1–NC) | 6.4 (3.9–8.3) |

Abbreviations: CI, confidence interval; NC, not calculated; OS, overall survival; PD, progressive disease; PFS, progression-free survival.

**Supplementary Figure S1.** Patient disposition.


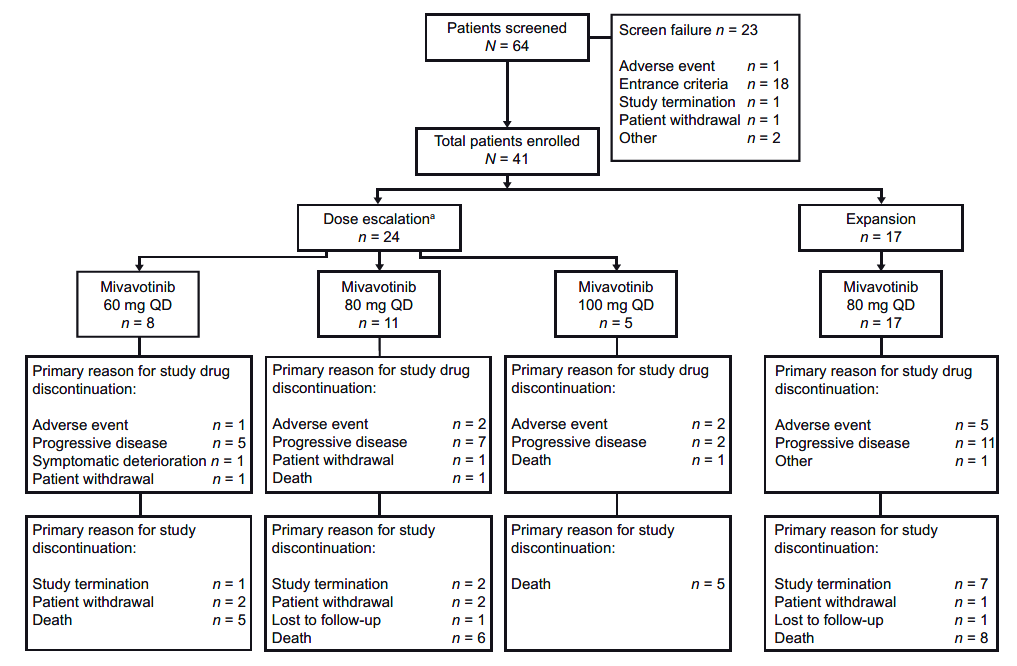


Abbreviations: QD, once daily.

^a^All patients in the dose-escalation phase treatment arms received mivavotinib in combination with nivolumab 3 mg/kg. In the expansion phase, 12 patients received mivavotinib at the determined recommended phase 2 dose (RP2D; 80 mg QD) plus nivolumab 3 mg/kg. Five patients received single-agent mivavotinib at the determined RP2D (80 mg QD) in weeks 1 and 2; from week 3 onwards, they received mivavotinib at the determined RP2D (80 mg QD) plus nivolumab 3 mg/kg.
